# Insights into spatial dynamics of the microbiome and resistome across the conventional and organic dairy farms

**DOI:** 10.1371/journal.pone.0352336

**Published:** 2026-06-25

**Authors:** Kabilan Mani, Vignesh Palanisamy, Bhuwan Shrestha, Zachariah Vice, Sushil Paudyal, Sapna Chitlapilly Dass

**Affiliations:** Department of Animal Science, Texas A&M University, College Station, Texas, United States of America; Universidade Catolica Portuguesa, PORTUGAL

## Abstract

Antimicrobial resistance (AMR) poses a serious global threat to human and animal health. While AMR has been reported in various environments, its distribution across different ecological compartments within dairy farms remains poorly characterized. In this study, we used large-scale shotgun metagenomic sequencing to characterize the microbiome and resistome across multiple sampling sites within one organic and one conventional dairy farm, including teats, liners, water troughs, feed area, milking parlour mats, bedding sand, and milk. Our results indicate that microbial community composition and resistance gene profiles were largely comparable between the two study farms, with sample type (ecological niche) exerting a stronger influence on community structure than farm management type. Pseudomonadota, Bacillota, and Actinomycetota were the dominant phyla, while Aerococcus, Glutamicibacter, and Pseudomonas were the most prevalent genera. Glycopeptide resistance genes were the most abundant ARG class, followed by lincosamide and tetracycline resistance genes. Milk samples exhibited a distinct microbiome and resistome composition compared to environmental samples. Strong correlations between microbiome structure, resistome profiles, virulence factors, and metal resistance genes were observed across farm niches, highlighting the interconnected nature of microbial communities and resistance elements across dairy farm environments. These findings provide foundational data for targeted surveillance and management strategies to mitigate antimicrobial resistance in dairy production systems.

## Introduction

The United States is one of the top-dairy (fluid-milk) producing countries in the world with a total production of 102.66 million metric tons (2024), underlining the importance of dairy industry’s contribution to the economy of the country. As of 2023, among the dairy farms located in the US, 21.6% were categorized into small (1–99 cows), while 23.4% were categorized into medium (100–499 cows), and 55% were categorized into large farms (500–2000 + cows), operating under either conventional or organic systems [1]. While conventional dairy farms have various options regarding stall bedding materials, housing (grazing or confined), feed composition, sanitizers in milking parlours, and antibiotic use, the organic farms must adhere to strict guidelines. The regulations pertaining to the creation and operation of organic farms have been guided by the National Organic Program (NOP), overseen by the US Department of Agriculture (USDA) and Agricultural Marketing Service (AMS). These guidelines regulate the methods, practices, and substances used in producing organic dairy products along with handling livestock within the farm. These guidelines further underline the significant differences in the operation of the farms between organic and conventional farms and these farm-specific procedures influence milk production and quality greatly. In the past two decades organic dairy farms have steadily increased owing to the increased consumer demands and the increased profitability in the operation of the organic dairy farms [2]. As of 2016, nearly 10% of the total milk produced in the USA has been produced from organic farms [3].

In dairy farming, microbiome composition and dynamics significantly influence milk quality and cow health. The term “microbiome” in this context not only relates to the rumen and skin-associated microbiota of cows, but extends to the microbial communities from feed, bedding materials, water, aerosols, and milking system components such as liners, together impacting milk quality and overall dairy herd health [4]. Apart from interacting with the beneficial microbes, dairy cows can also acquire pathogenic organisms from their environment, potentially contaminating milk and posing food security risks [5,6]. Given the extensive microbial interactions cows undergo within farm settings, it is important to characterize microbiome composition across various farm environments, regardless of whether operations are conventional or organic. Further, these characterizations are especially important in the organic farm settings, since the usage of antimicrobials is not permitted, increased interactions of beneficial commensal microbiota with the cows would help prevent the growth of pathogenic microbes.

AMR has emerged as a significant global threat to the health of both humans and animals and is a major contributor to global mortality, accounting for approximately 9% of all deaths worldwide [7]. The emergence and spread of AMR are driven by complex interactions at the intersection of human, animal, and environmental systems, facilitating the transmission and persistence of resistant microorganisms [8]. Although AMR occurs naturally, the widespread and often excessive use of antibiotics in healthcare, agriculture, and related sectors has led to significant antibiotic pollution. This, in turn, has been linked to the increased prevalence and transmission of microorganisms harboring ARGs across diverse environments [9]. The presence of ARGs and ARG harbouring bacteria in food-producing animals, has raised significant public health concerns. These factors contribute to potential transmission pathways for AMR bacteria between animals and humans, posing a risk to consumer safety and public health [10–12]. Several studies have detected ARGs across dairy farm environments, including bedding, feed, equipment, water troughs, soil, wastewater, and raw milk, with cattle feces and manure being the primary reservoirs [13–16]. ARGs found in dairy cattle are often associated with mobile genetic elements (MGEs), indicating that horizontal gene transfer might play a key role in spreading resistance genes both within bacterial populations and between cows and their surrounding environment. Along with the exposure to the antimicrobial substances, microbial communities in dairy farms are also subjected to biocides and heavy metals, further increasing the likelihood of acquiring resistance traits. The major use of antimicrobial in the conventional farms are directed towards treating clinical conditions like mastitis, metritis, retained placenta, bacterial diarrhoea and respiratory infections, with mastitis being the leading cause [17,18].

Given the major role played by the microbial community and its associated resistome in dairy farms, it is important to understand the difference in the microbiome and resistome structure in the conventional and organic farms. Though several studies have been conducted on the investigation of bacterial diversity in dairy farms, these studies have either focused on a specific site like bedding material or specific samples like milk or manure [19–21]. There is lack of studies comparing the microbiome and resistome diversity between the conventional and organic farms, especially tracking the bacterial movement from sand to milk. Most of the available studies on comparing the same have been carried out through culture-dependent techniques, lacking a broader and in-depth analysis [13,15]. We used shotgun metagenomic sequencing across organic and conventional dairy farms to investigate the impact of farm management on microbiome and resistome structures, to understand the associations with virulence factors, integrons, and metal resistance genes, and evaluate the mobility of ARGs via their plasmid distribution. Conventional farms often use antibiotics more extensively, creating greater selective pressure for the emergence and persistence of ARGs. In contrast, organic farms follow strict regulations that restrict or prohibit antibiotic use, which might result in distinctly different microbiome and resistome profiles. Based on these differing practices, we hypothesize that the distribution of bacterial communities and ARGs will differ significantly between conventional and organic farms, mainly due to the limited antibiotic use in organic systems. In the United States, organic dairy farming is governed by the National Organic Program (NOP), administered by the USDA Agricultural Marketing Service (USDA/AMS). Organic certification requires compliance with strict production standards, including the prohibition of antibiotic use for disease treatment, mandatory access to pasture for a minimum of 120 days per year, and at least 30% of dry matter intake from pasture during the grazing season (https://www.ams.usda.gov/about-ams/programs-offices/national-organic-program).

## Results

In this study we examined the distribution patterns of microbiome and resistome among the organic and conventional dairy farms. Further, we explored the associations between the microbiome and the ARGs, integrons, and metal resistance genes.

### Description of the metagenomic data

A total of 171 samples were collected for this study, comprising 77 from a conventional farm and 94 from an organic farm (S1 Table). Shotgun metagenomic sequencing generated approximately 1 terabase (Tb) of high-quality data, with an average of 3 gigabases (Gb) per sample. Approximately 7–10% of reads were removed after quality filtering and host-read removal in the non-milking area samples, while liner and teat samples showed an average removal of 15%, and milk samples had about 40% of reads removed on average. After quality filtering, de novo assembly, and gene prediction, 35,438,842 and 33,286,346 open reading frames (ORFs) were identified from the conventional and organic farm samples, respectively. Clustering these ORFs at 90% identity yielded 14,524,789 representative sequences for the conventional farm and 16,488,952 for the organic farm, indicating a slightly higher gene diversity in the organic farm samples.

### Microbiome structure of dairy farms

Alpha diversity analysis revealed that, when all sample types were pooled, samples from the conventional dairy farm exhibited slightly higher Shannon diversity indices compared to those from the organic farm. However, this overall pattern was not consistent across individual sample types. Per-sample-type Wilcoxon rank-sum tests showed significant differences in Shannon diversity between farm types in two of the eight sample types: feed area (conventional: 3.60 ± 0.36 vs. organic: 2.72 ± 0.20; p = 4.1 x 10 ^−5^) and water troughs (conventional: 4.65 ± 0.74 vs. organic: 3.33 ± 0.53; p = 4.4 x 10 ^−4^), with conventional farm samples showing higher diversity in both cases. The remaining six sample types showed no significant differences in Shannon diversity (liner: p = 0.374; mat: p = 0.310; milk: p = 0.928; sand: p = 0.365; teats after dipping: p = 0.674; teats before dipping: p = 0.923). Among all sample types, milk had the lowest Shannon diversity, while sand showed the highest (S1,S2 Figs). Linear mixed-effects modeling (lme4 in R) with farm type as a fixed effect and sample type as a random intercept confirmed a significant overall farm-type effect on Shannon diversity (estimate = −0.49, p = 0.0007), indicating that organic farm samples had lower Shannon diversity after accounting for differences between sample types. The intraclass correlation coefficient (ICC) was 0.60, indicating that 60% of the total variance in Shannon diversity is attributable to differences between sample types, with farm type accounting for a smaller but statistically detectable proportion.

**Fig 1:**
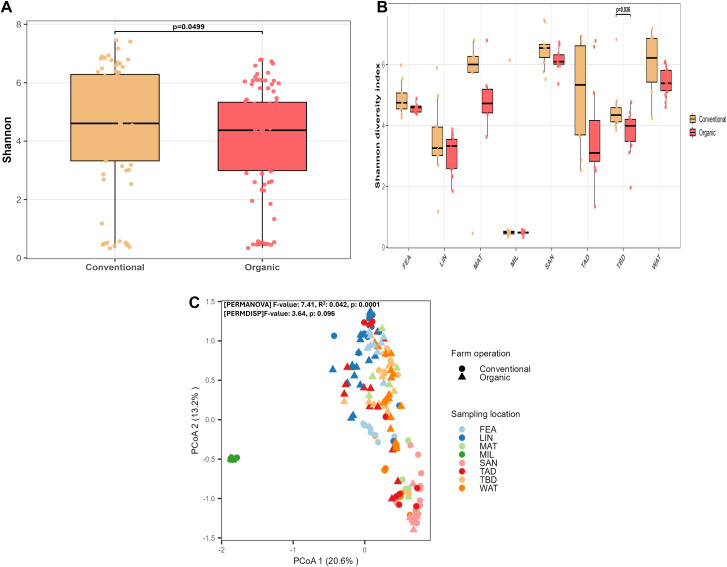
A. Shannon diversity index values of the microbiome structure prevalent in conventional and organic dairy farms (*p < 0.05). **B.** Shannon diversity measure of the microbiome distribution observed among various samples collected from organic and conventional farms. **C**. Principal Coordinates Analysis (PCoA) based on Bray-Curtis distances illustrating the microbiome distribution across samples collected from organic and conventional farms. Differences between groups were assessed using PERMANOVA. Only significant P-values alone are displayed. Sample types include FEA – Feed area, LIN – Liners, MAT – Mat, MIL – Milk, SAN – Sand, TAD – Teats after iodine dipping, TBD – Teats before iodine dipping, and WAT – Water troughs.

Microbial community composition was assessed using PCoA based on Bray-Curtis dissimilarity. PERMANOVA analysis of all 171 samples revealed a statistically significant but minor effect of farm type on community composition (F = 7.41, R² = 0.042, p = 0.001), explaining only 4.2% of the total variation. Milk samples formed a distinct cluster separate from all other sample types (Fig 1C). To assess whether this distinct clustering drove the significant PERMANOVA result, we re-ran the analysis excluding milk samples (n = 148). The farm-type effect remained statistically significant but minor (F = 7.40, R² = 0.048, p = 0.001), explaining 4.8% of the total variation. When both sampling location and farm type were included as factors, sampling location explained 30.7% of community variation (F = 11.12, R² = 0.307, p = 0.001), approximately six times more than farm type (R² = 0.049, F = 10.72, p = 0.001). PERMDISP analysis showed no significant difference in within-group dispersion between farm types when all samples were included (F = 3.64, p = 0.096); however, after excluding milk samples, PERMDISP detected significant differences in dispersion (F = 6.93, p = 0.012), indicating that some of the observed PERMANOVA effect may reflect differences in within-group variability rather than centroid position alone. These results indicate that ecological niche exerts a substantially stronger influence on microbiome composition than farm management type.

Taxonomic profiling showed bacterial reads dominated across all samples, followed by Archaea and Fungi. Across the samples, 37 phyla (including 4 candidate phyla), 97 classes, 201 orders, 1958 genera, and 6498 species were detected. Pseudomonadota, Bacillota, and Actinomycetota were the dominant phyla (S3 Fig). We found that conventional farms were dominated by Bacillota, whereas organic farms showed higher proportions of Actinomycetota. Among the individual samples, Bacillota dominated milk while Actinomycetota dominated the sand samples. However, significant differences were observed in mat and feed area samples (Wilcoxon rank-sum test, FDR-adjusted p < 0.05), with conventional farms dominated by Bacillota and organic farms by Pseudomonadota.

**Fig 2:**
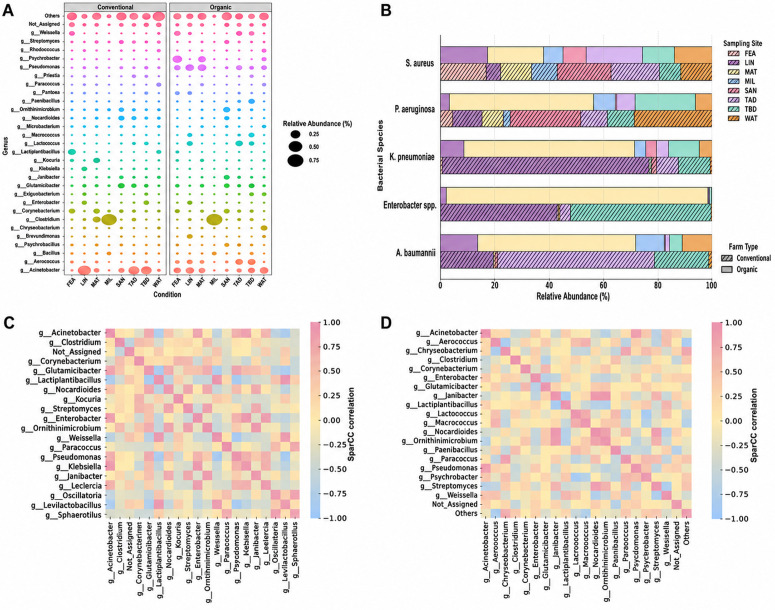
A. Bubble plot illustrating the taxonomic distribution of sequencing reads from organic and conventional farm samples, aggregated at the genus level. **B**. Bar plot showing the prevalence of ESKAPE pathogens in sampling sites from organic and conventional farms. Correlation network analysis illustrating genus-level positive and negative associations among microbiome samples from **(C)** conventional and **(D)** organic dairy farms. Sample types include FEA – Feed area, LIN – Liners, MAT – Mat, MIL – Milk, SAN – Sand, TAD – Teats after iodine dipping, TBD – Teats before iodine dipping, and WAT – Water troughs.

At the genus level, 1958 bacterial genera were detected across all samples, with *Acinetobacter* as the dominant genus across both farm types, followed by *Aerococcus*, *Glutamicibacter*, *Pseudomonas, Streptomyces, Corynebacterium, Janibacter,* and *Weissella* (Fig 2A). *Clostridium* was the dominant genera in the milk samples from both operations (>85%), indicating its niche adaptation. Teat swabs from organic farms exhibited greater genus-level diversity than those from conventional farms, despite the usage of iodine disinfection, which caused no major shifts in overall community structure. The taxonomic compositional similarity between teat and liner samples indicates microbial exchange between these surfaces.

ESKAPE pathogens were more prevalent in conventional farms, with over twice the number of taxonomic reads compared to organic farms. While teats and liners represented major reservoirs of these pathogens among all the samples studied, *Acinetobacter baumannii* and *Enterobacter* spp. were the dominant bacterial taxa among the ESKAPE pathogens. Interestingly, although iodine dipping did not alter overall microbiome composition of teats, it was active against ESKAPE organisms, suggesting a targeted antimicrobial effect (Fig 2B).

Core microbiome analysis was conducted using thresholds of ≥0.1% relative abundance and ≥10% prevalence. These thresholds were selected to capture taxa consistently present across samples while accommodating the ecological heterogeneity inherent in multi-niche sampling designs, consistent with approaches used in comparable environmental metagenomics studies (Risely, 2020; Shade and Handelsman, 2012). The analysis identified *Acinetobacter* and *Clostridium* as universal core members across farms, with *Glutamicibacter* and *Lactiplantibacillus* specific to conventional samples, and *Aerococcus*, *Lactococcus*, *Pseudomonas*, and *Psychrobacter* specific to organic farms. Using a log LDA score threshold of 4, LEfSe analysis identified 131 genera as significantly different between farm types, with a strong asymmetry: 130 genera enriched in conventional samples and only *Carnobacterium* enriched in organic samples. To validate these findings, we performed ANCOM-BC analysis (Lin and Peddada, 2020), a compositionality-aware method that accounts for sampling fraction bias. ANCOM-BC analysis of 2,710 genera identified 828 as differentially abundant (q < 0.05), with a near-equal distribution: 416 genera enriched in organic farm samples and 412 enriched in conventional farm samples (S10 and S11 Figs). Among the most differentially abundant genera, *Carnobacterium* (log_2_FC = +3.41, q = 2.7 x 10 ^−10^), *Psychrobacter* (log_2_FC = +3.11), and *Aerococcus* (log_2_FC = +2.84) were most enriched in organic samples, while *Thermoactinomyces* (log_2_FC = −4.10, q = 8.9 x 10 ^−21^), *Thermobifida* (log2FC = −3.62), and *Symbiobacterium* (log_2_FC = −2.66) were most enriched in conventional samples. The balanced distribution of differentially abundant genera identified by ANCOM-BC contrasts with the asymmetric LEfSe results, indicating that the original LEfSe asymmetry reflected methodological sensitivity to compositionality rather than a true biological imbalance. Per-sample-type ANCOM-BC analyses revealed that the number of differentially abundant genera varied across ecological niches, with feed area and water troughs showing the most differences and mat samples the fewest.

Correlation network analysis using SparCC (p < 0.01) at a correlation threshold of 0.6 among the top 30 genera revealed both positive and negative interactions within the bacterial communities across farm samples (Fig 2 C and D). The organic farm samples exhibited a higher proportion of positive associations among bacterial genera. Genera such as *Lactiplantibacillus*, *Weissella*, and *Janibacter* formed multiple positive correlations with other taxa, suggesting potential cross-feeding or syntrophic interactions. In contrast, the conventional farm network displayed comparatively more negative correlations, particularly among *Pseudomonas*, *Enterobacter*, and *Klebsiella*. Additionally, UpSet Venn plots showed that nearly all genera were shared across sampling sites within each farm type, suggesting widespread microbiome exchange between different niches (S5 Fig).

### Resistome structure of the Dairy farms

Analysis of ARGs revealed 5042 gene variants across 393 AMR gene families in organic farm samples. These were further categorized into 120 drug families and seven resistance mechanisms. In comparison, conventional farm samples contained 5304 gene variants grouped under 396 AMR gene families, distributed across 121 drug families and the same seven resistance mechanisms.

The overall ARG to bacteria ratio, estimated from the ARG/16S rRNA gene ratio, was slightly higher in conventional (15.46) than in organic farms (12.72). Among sample types, sand exhibited the highest ratios:105.09 in conventional and 90.54 in organic farms,while milk had the lowest, at 0.32 and 0.44, respectively.

**Fig 3:**
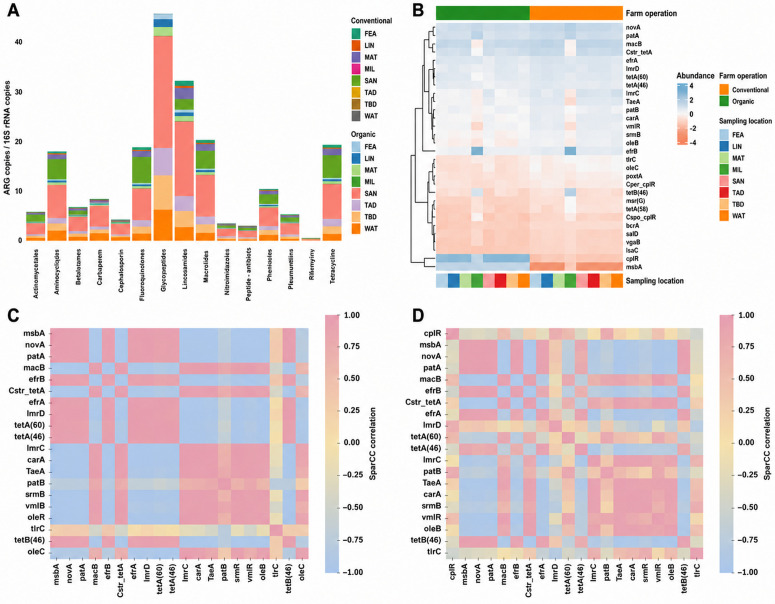
A. Bar plot showing the normalised abundance of antimicrobial resistance genes (ARGs) grouped by drug class in organic and conventional dairy farm samples. **B**. Heat map illustrating the distribution of antimicrobial resistance genes (ARGs) at the gene level across samples collected from organic and conventional dairy farms. Correlation network analysis illustrating genus-level positive and negative associations among ARGs from **(C)** conventional and **(D)** organic dairy farms. Sample types include FEA – Feed area, LIN – Liners, MAT – Mat, MIL – Milk, SAN – Sand, TAD – Teats after iodine dipping, TBD – Teats before iodine dipping, and WAT – Water troughs.

Across the samples, glycopeptide resistance genes were the most prevalent, followed by lincosamide, tetracycline, macrolide, aminoglycoside, carbapenem, and fluoroquinolone classes (Fig 3A). Conventional farms generally exhibited higher ARG abundances across these drug categories than organic farms. Relative abundance (R.A.) of ARG hits was analyzed at four CARD-defined levels. At the resistance mechanism level (S6 Fig), antibiotic efflux and target protection dominated (>75% of ARG reads), followed by target alteration, inactivation, and replacement, a trend consistent across all sample types. At drug class level, the R.A. (S7 Fig) were similar between farm types, with lincosamide and tetracycline resistance genes being most abundant. Milk samples, however, displayed a distinct dominance of tetracycline resistance followed by lincosamide, macrolide, and cephalosporin.

At the gene family level, ATP-binding cassette (ABC) antibiotic efflux pump related reads was most prevalent in both farm types (>20%), followed by ABC-F ribosomal protection proteins, resistance-nodulation-division (RND) transporters, and major facilitator superfamily (MFS) efflux systems. Organic samples showed slightly higher representation of van ligase–associated genes, while milk samples again displayed a distinct resistance profile enriched in tetracycline-related ribosomal protection proteins. At the gene level (Fig 3B), *cplR*, an ABC-F ATPase conferring pleuromutilin and lincosamide resistance and *msbB* were abundant in organic farm samples. Other highly represented genes (*msbA, novA, patA, cstR_tetA*, and *macB*) were observed in both farm types, whereas milk samples were particularly enriched with *efrB* and *tetB* (46).

Core resistome analysis (>1% relative abundance; > 80% prevalence) revealed higher levels of core ARGs in conventional farms compared to organic farms. They included *oleC, tetB(46), tetA(58), poxtA, cplR, efrB*, and *msr(G)* while *cplR* and *msbA* were exclusive to organic samples. LEfSe analysis (LDA > 4) did not identify significant enrichments in conventional farms however highlighted fluoroquinolone (*efrA, patB, arlR*) and streptogramin related (*lsaA*) genes as markers in organic farms (S8 Fig).

UpSet plots demonstrated resistome gene hits overlap between farm types, with 198 and 182 shared ARGs in conventional and organic samples, respectively (S9 Fig). Water harboured the highest number of unique ARGs in both farms (179 and 299 in organic and conventional farms, respectively), underscoring its role as a potential reservoir. Like microbial interactions, organic farm showed more frequent positive correlations with positive associations among efflux-related genes like *msbA*, *patA*, *efrA*, *lmrC*, and *vmrA* (Fig 3C and D).ARG risk ranking analysis indicated the prevalence of high-risk resistance genes across the farm samples. Both farm types showed a similar distribution of risk profiles. Among the conventional farm samples, approximately 1.5% of the ARGs belonged to the Risk-I group, while 1.6% and 5% were classified under the Risk-II and Risk-III groups, respectively. Similarly, in the organic farm samples, the Risk-I and Risk-II groups each comprised 1.6% of the ARGs, while the Risk-III group accounted for 5.2%.

**Fig 4:**
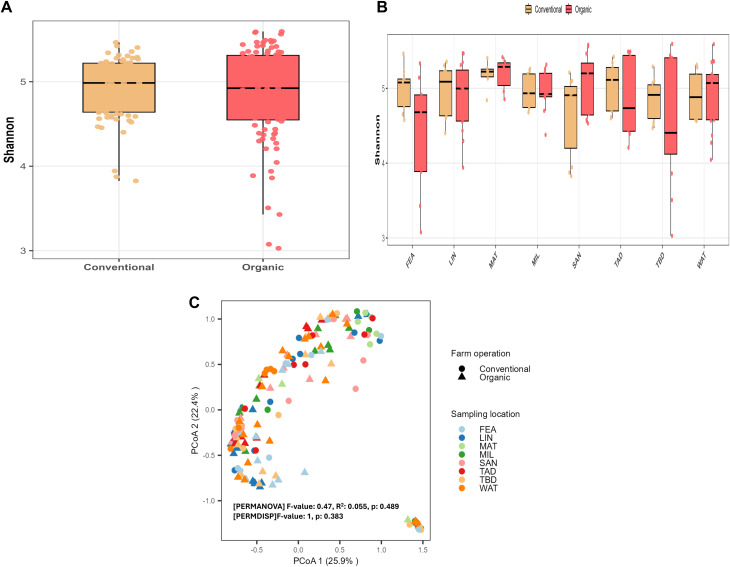
A. Shannon diversity index values of the resistome structure prevalent in conventional and organic dairy farms (*p < 0.05). **B**. Shannon diversity measure of the resistome distribution observed among various samples collected from organic and conventional farms. **C.** Principal Coordinates Analysis (PCoA) based on Bray-Curtis distances illustrating the ARGs distribution across samples collected from organic and conventional farms. Differences between groups were assessed using PERMANOVA. Only significant P-values alone are displayed. Sample types include FEA – Feed area, LIN – Liners, MAT – Mat, MIL – Milk, SAN – Sand, TAD – Teats after iodine dipping, TBD – Teats before iodine dipping, and WAT – Water troughs.

Shannon diversity analysis of the resistome genes indicated no significant difference between the conventional and organic samples (p-value > 0.05) (Fig 4A). When alpha diversity values were compared across various sample types, similar Shannon diversity indices were observed, suggesting a comparable distribution of the resistome (Fig 4B). Resistome composition relatedness among the samples was analyzed using PCoA plots derived from Bray–Curtis dissimilarity matrices and further validated statistically through PERMANOVA (F-value: 0.47; R-squared: 0.055; p-value = 0.489). The clustering patterns showed that samples from different locations within both organic and conventional farms tended to cluster together. Similarly, PERMDISP (F = 1, p = 0.383) confirms that there is no significant difference in within-group dispersion, (Fig 4C). As with the microbiome analysis, pooling all sample types under farm-level categories for resistome diversity comparisons may introduce confounding by sample matrix.

Overall, the diversity analysis indicated that farming operational procedures did not significantly influence the resistome distribution patterns between organic and conventional dairy farms, mirroring observations made in microbiome distribution.

### Drivers of ARGs within the dairy farms

We next analysed the prevalence of MGEs that facilitate the ARG transmission across farm environments (S2–S4 Tables). Analysis against BacMet database revealed the presence of abundant metal-resistant transporters associated with copper (*copA*), molybdenum (*wtpC*), zinc (*znuC, troB*), and iron (*smrA*) (>3% R.A.) (S2 Table). Analysis against Integron further indicated the high prevalence of the general integrase (*intI*), transposase-related genes (*tnp, tnpA, tnpA.1*) along with chloramphenicol acetyltransferase genes (*catB, cmlA*) underscoring the potential for horizontal ARG transfer.

In contrast, a similar analysis for insertion sequences (IS elements) showed that their overall abundance was relatively low (<1% R. A.) compared to other MGEs (S3 Table) with *ISAba1*, *IS15*, *IS2*, and *ISBce1* being the most prevalent. Sequence similarity search against virulence factor database (S4 Table) revealed *iroC*, encoding a salmochelin export protein, as the dominant virulence factor across both farm types. Other frequently detected virulence-associated genes included *hlyB* (hemolysin secretion), *ybtP/Q* (yersiniabactin transport), and *rtxB* (RTX toxin secretion). Notably, *clbP*, involved in colibactin activation, showed exceptionally high abundance (>24%) in feed area samples from the organic farm, indicating site-specific enrichment of toxin-related functions.

**Fig 5:**
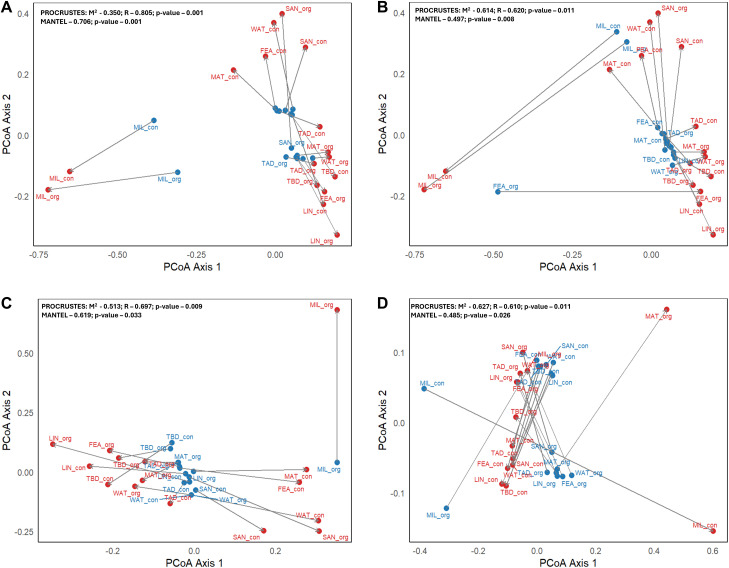
Procrustes analysis illustrating the correlation between the overall structure of microbiome and resistome (A), between the overall structure of microbiome and virulence factors (B), between the overall structure of microbiome and metal resistance genes (C), and between the overall structure of ARGs and integrons (D) in samples collected from dairy farms. Blue = conventional farm; green = organic farm. FEA – Feed area; LIN – Liners; MAT – Mat; MIL – Milk; SAN – Sand; TAD – Teats after iodine dipping; TBD – Teats before iodine dipping; WAT – Water troughs.

Correlation analyses demonstrated strong associations between microbial community composition and resistome structure. Procrustes analysis confirmed a significant relationship between the microbiome and ARG profiles (M² = 0.350, *r* = 0.805, *p* = 0.001), further supported by the Mantel test (*r* = 0.706, *p* = 0.001) (Fig 5A–D). The microbiome also correlated with virulence factors (Procrustes *r* = 0.620, *p* = 0.011; Mantel *r* = 0.497, *p* = 0.008) and with metal-resistance genes (Procrustes *r* = 0.697, *p* = 0.009; Mantel *r* = 0.619, *p* = 0.033). Weak correlations between ARGs and integrons (Procrustes *r* = 0.610, *p* = 0.011; Mantel *r* = 0.485, *p* = 0.026) suggest that horizontal gene transfer mechanisms partly shape resistome architecture within dairy farm microbiomes.

### Plasmidome structure of the Dairy Farms

We conducted an analysis to determine the percentage of antimicrobial resistance genes (ARGs) carried within plasmids due to their potential role in facilitating horizontal gene transfer across various organisms. Our analysis indicated that, among conventional dairy farms, 28% of the ARGs were plasmid-borne, while in the organic farm samples, 26% of the ARGs were plasmid-borne.

**Fig 6:**
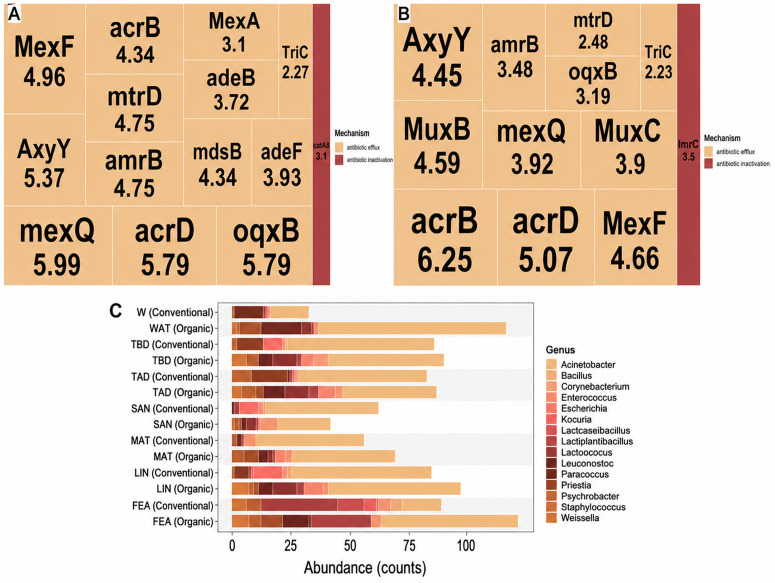
Tree map illustrating the distribution of ARGs identified within plasmids, categorized by resistance mechanism, gene family, and gene level, in samples from (A) organic and (B) conventional dairy farms. C. Bar plots illustrating the genus-level taxonomic profiles of microbiomes in samples collected from organic and conventional dairy farms. FEA -Feed area, LIN – Liners, MAT- Mat, MIL – Milk, SAN – Sand, TAD – Teats after iodine dipping, TBD – Teats before iodine dipping, WAT – Water troughs. Due to their low microbial diversity, milk samples were excluded from the plasmid analysis.

Plasmid-associated ARGs were predominantly linked to antibiotic efflux mechanisms in both farm types, accounting for 71% and 74% of total ARG reads in organic and conventional farms, respectively (Fig 6A-B). Antibiotic inactivation (20%) and target alteration (13%) were the most prevalent secondary mechanisms of antibiotic resistance. At the gene-family level, RND transporters were dominant (>60%), followed by major facilitator superfamily (MFS) efflux pumps and chloramphenicol acetyltransferases. Among conventional farm samples, MCR-type phosphoethanolamine transferases were dominant. The most prevalent efflux genes across both farm types included *acrD*, *mexF*, *mexQ*, *oqxB*, *AxyY*, *amrB*, and *acrB*, highlighting multidrug resistance potential mediated by membrane-associated transport systems.

Taxonomic profiling of plasmid reads revealed *Acinetobacter* as the predominant plasmid-harboring genus in both farm types (Fig 6C). Among the plasmids identified from the individual samples, *Lactiplantibacillus* dominated feed area plasmids, *Paracoccus* was dominant in water, while *Lactococcus* and *Priestia* was dominant in teat and liner samples from organic and conventional farms, respectively.

## Discussion

In this study, we employed shotgun metagenomics to characterize the spatial distribution of the microbiome and resistome across multiple ecological compartments within two dairy farms operating under organic and conventional management. Beyond comparing farm management types, this study contributes to the broader understanding of microbial dynamics, antimicrobial resistance gene distribution, and the interconnectedness of different farm niches as potential reservoirs and conduits for microbial exchange. Understanding these dynamics is crucial, as farm management practices can significantly shape microbial ecosystems, potentially impacting cattle health and the safety of dairy products. While previous research has acknowledged the role of farming practices in influencing microbial communities, a comprehensive analysis contrasting the microbiome and resistome profiles between organic and conventional dairy farms remains limited. Our findings contribute to this knowledge gap, offering insights into the ecological consequences of agricultural practices on microbial and resistance gene distributions within dairy farm. Consistent with previous studies comparing organic and conventional dairy systems (Pitta et al., 2020; Rovira et al., 2019), our investigation found that microbial community differences were minimal between the two study farms, suggesting that factors other than management type may exert greater influence on microbial assembly within these specific farm environments. Specifically, PERMANOVA analysis demonstrated that sampling location (ecological niche) explained 30.7% of community variation compared to only 4.6% attributable to farm type, and per-sample-type alpha diversity comparisons revealed no significant differences in Shannon diversity between farm types within seven of the eight sample types examined (Mann-Whitney U, all p > 0.05), while significant differences were detected in feed area (p = 4.1 x 10 ^−5^) and water troughs (p = 4.4 x 10 ^−4^). Linear mixed-effects modeling confirmed a significant overall farm-type effect on Shannon diversity (p = 0.0007), with 60% of variance attributable to sample type (ICC = 0.60). However, the resistome composition remained largely similar, suggesting that ARGs are functionally conserved despite taxonomic shifts. The persistence of similar ARG profiles across both farm types likely reflects multiple interacting factors. Horizontal gene transfer via mobile genetic elements may contribute to the maintenance of the ARG pool across environments; however, the duration of organic management is a critical variable, as bacteria can lose MGEs carrying resistance determinants due to fitness costs in the absence of antibiotic selection pressure (Starikova et al., 2013). Co-selection mechanisms, including the use of heavy metals as feed additives, may also maintain resistance determinants independently of direct antibiotic pressure. The persistence of similar resistance genes in taxonomically distinct communities reflects the functional redundancy of the resistome, highlighting its resilience and widespread dissemination across farm systems. [13,15]. Similarly, another study comparing the microbiomes of two geographically distant dairy farms found a comparable pattern, reinforcing the idea that factors other than farm operation may exert greater influence on microbial distribution [16].

Contrary to previous studies, we observed minimal intra-sample variability in microbiome and resistome composition across our datasets. This observation likely reflects both the relative homogeneity of the sample types selected and the fact that all samples within each farm type originate from a single farm. Sampling from multiple farms of each management type would be expected to introduce greater variability, as each farm represents a unique combination of geographic location, herd genetics, and management history. Highly heterogeneous samples, such as manure and cow feces, are known to harbour diverse microbiomes, while environmental samples, like those from the feed area or sand used as bedding, tend to exhibit less variability [16]. In our study, the samples selected were of a similar type, which likely contributed to the consistent microbial profiles observed.

Our analysis revealed that while the dominant bacterial phyla Bacillota and Pseudomonadota align with previously documented patterns in dairy farm environments [13,15,16], notable differences emerged at the genus level. While dominant genera such as *Aerococcus*, *Glutamicibacter*, *Pseudomonas*, *Streptomyces*, *Corynebacterium*, *Janibacter*, and *Weissella* have been reported in other dairy farm microbiome studies, our study identified a higher abundance *Glutamicibacter*, *Janibacter*, and *Weissella* in our samples. We hypothesize that the frequent animal movement across various farm locations may contribute significantly to the increased detection of bacterial genera typically considered rare or niche specific. This was particularly evident for *Janibacter* and *Glutamicibacter*. Specifically, *Janibacter*, an actinomycete typically associated with soil and other environmental niches [22], was notably detected within the teat microbiome in our study. Similarly, *Glutamicibacter*, generally considered a member of the bovine microbiome [23], was found in locations such as the feed area and sand bedding. These findings suggest a continuous microbial flux between various farm environments. The metabolic versatility and intrinsic resistance to environmental stressors, which may further explain their adaptability and persistence across diverse farm locations.

This study allowed us to investigate the microbiome composition of specific ecological niches of dairy farms, providing valuable insights into potential impacts on cattle health and dairy product quality. Among these niches, bedding material is particularly significant, as dairy cows spend a substantial portion of their daily activities (10–13 hours per day) in contact with these substrates. Both dairy farms in our study utilized sand as bedding material, a material previously associated with lower bacterial loads relative to organic substrates [24]. Interestingly, certain bacterial genera such as *Ornithinimicrobium*, *Nocardioides*, and *Aerococcus* were predominantly found in sand samples. However, these genera were also detected as dominant members of the teat microbiome and, to a lesser extent, the liner microbiome, especially in samples from the organic farm. *Aerococcus*, a Gram-positive bacterium from the phylum *Bacillota* commonly found in soil, was likely transmitted from sand to liners via the teats, illustrating a clear chain of microbial transmission between closely connected farm environments [25]. Similarly, *Nocardioides* was abundant in both organic and conventional farm samples, although previous studies have identified it as a signature genus associated with organic dairy products [26]. These findings emphasize the interconnectedness of different farm microenvironments and highlight how microbial communities can disperse across them.

Microbiome analysis revealed *Acinetobacter* as the dominant genus across all samples, a resilient and opportunistic pathogen commonly found in dairy farm environments. Its presence is often linked to contamination from residual water in milking equipment, inadequate cleaning, and poor milk handling practices. Known for withstanding desiccation and disinfectants like phenols and chlorhexidine, *Acinetobacter*’s high abundance, coupled with rising multidrug resistance, underscores the need for improved microbial surveillance and strengthened biosecurity measures [27–29]. Notably, we detected an unexpectedly high abundance of *Clostridium* in milk samples from both dairy farms. Although *Clostridium* is frequently identified in raw milk, its presence at such elevated levels is rarely documented. The increased prevalence of this spore-forming bacterium highlights potential risks for dairy product spoilage, particularly in cheese production [14,30]. Additionally, our findings align with existing literature suggesting that iodine-based teat disinfection does not significantly alter the overall teat microbiome but selectively targets certain bacterial groups, such as *Streptococcus*. Similarly, we observed a reduction in the ESKAPE group of pathogens following treatment, although no significant changes were noted in overall microbial diversity on the teats [31,32].

Although antibiotics are not used in organic farms, the detection of ARGs highlights the natural prevalence of resistance in the environment. ARGs are known to be widely distributed in nature and may be maintained due to their co-localization in complex resistance clusters that include genes conferring resistance not only to antibiotics, but also to metals and biocides. Furthermore, the inclusion of heavy metals such as zinc and copper in the diets of organically raised cattle, as alternatives to antibiotics, may promote the emergence of bacterial populations co-resistant to both metals and antibiotics. Notably, the identification of ARGs associated with antibiotics that were never used in the conventional farms may suggest the occurrence of co-selection, or the enrichment of microbial communities that naturally harbour these resistance genes [15].

Previous studies examining the ARG profiles of dairy farms have often reported significant variation in resistome structures among samples collected from different locations within the same farm [10,16,20]. In contrast, our study revealed a relatively uniform distribution of ARGs across samples from both organic and conventional farms. This consistency may be attributed to the homogeneity of the sampling sites selected. Other studies typically included more heterogeneous sample types, such as effluent, cow dung, and manure, which likely contributed to the observed variation in resistome profiles. Notably, although sand is generally considered an inorganic bedding material with low bacterial load, we found the highest ARG/16S rRNA ratio in the sand samples. This is likely due to the use of recycled sand, which can accumulate organic matter over time, such as manure, urine, and undigested feed, thereby enriching both microbial communities and ARG abundance [24,33].

In the United States, various antibiotics are used to treat common dairy cow infections—including mastitis, respiratory and uterine infections, and foot disease—such as β-lactams (penicillin, cefapirin, ceftiofur, amoxicillin, hetacillin, cloxacillin), macrolides (erythromycin, tylosin, tilmicosin), coumarins (novobiocin), lincosamides (pirlimycin, lincomycin), tetracyclines, sulfonamides, and florfenicol [34]. Several studies have identified dairy farms as hotspots for ARGs, with patterns in our study aligning with previous reports from both organic and conventional systems, highlighting resistance to tetracyclines, macrolides, lincosamides, and aminocoumarins [13,35–37]. Notably, our study is the first to report glycopeptides as the dominant ARG class in dairy farms, primarily due to vancomycin resistance variants. This is alarming, as vancomycin is a last-resort antibiotic in human medicine, and its resistance raises concerns about potential zoonotic transmission. Supporting this, vancomycin-resistant *Staphylococcus aureus* has been isolated from milk samples [38,39]

Furthermore, the distinct AMR profile observed in the milk samples indicated that most of the resistance genes detected in the milk were different from those present in the environmental samples, suggesting a unique pattern of gene dissemination within the farm system. Although Alexa et al. (2020) reported similar AMR profiles between farm environments and milk, our findings revealed a clearly distinct AMR gene composition in the milk samples compared to the farm environment [40]. The *efrB* gene encodes one subunit of the EfrAB ATP-binding cassette (ABC) multidrug efflux pump, first characterized in *Enterococcus faecalis* strain V583. The EfrAB complex, comprising the EfrA and EfrB subunits, confers broad-spectrum resistance to several structurally unrelated antimicrobials and dyes, including ethidium bromide, acriflavine, and certain fluoroquinolones, through an ATP-driven efflux mechanism that actively exports toxic compounds out of the cell [41]. Beyond *Enterococcus*, *efrA* and *efrB* genes have been reported in multiple Gram-positive genera, highlighting their wider ecological dissemination [42]. Investigations into virulence and antibiotic-resistance factors in *E. faecalis* associated with streptococcosis in aquaculture species have confirmed the occurrence of the *efrB* gene, indicating its involvement in multidrug resistance under diverse environmental conditions [43]. Similarly, *tetB*(46) aprt of *tetAB*(46), a predicted heterodimeric ABC transporter conferring tetracycline resistance was first identified in *Streptococcus australis* isolated from human oral cavity [44]. Since then, it has been reported in diverse environmental samples like silage associated with *Lactocaesibacillus* sp. and among sewage samples [45,46]. These results indicate that though these AMR genes are not categorised as high-risk antibiotics, their presence in both farms could indicate their emergence as a potential high-risk AMR gene.

Organic farms were exclusively found to contain *cplR* and *msbB* gene. *cplR* gene is a genome-encoded resistance determinant found primarily in Gram-positive bacteria, notably in species like *Clostridium perfringens* and *Clostridioides difficile*. It confers intrinsic resistance to PLSA antibiotics, and its resistance effect can be significantly enhanced when it is found alongside other resistance genes like *cat*, *emr*, or *bmr* genes [47]. The dominance of *Clostridium* sp. related reads in the conventional farms could be attributed to the prevalence of *cplR* related genes in the organic farm environments. Similarly, the gene *msbB* is a core chromosomal gene found in Gram-negative bacteria like *Escherichia coli* and *Salmonella*, where its product is an enzyme involved in the final myristoylation step of lipid A synthesis leading to the reduced binding of polymyxin-class antimicrobials [48]. The higher prevalence of Gram-negative organisms in the organic farm environments could be attributed to the higher prevalence of *msbB* gene. Beyond the above-mentioned details, *cpl*R and *msb*B has broader ecological roles beyond conferring antimicrobial resistance. Without the use of antibiotics in the organic farms, there is lesser selection pressure for these intrinsic AMR genes, enabling them to prevail in these environments.

Though the presence of high-risk antibiotics (rank-I) were minimal (< 1.5% R.A.), they were found exclusively associated as part of conventional farm samples core microbiome. The *poxtA* gene encodes an ATP-binding cassette F (ABC-F) ribosomal protection protein that confers reduced susceptibility to phenicols, oxazolidinones, and tetracyclines through ribosome shielding rather than efflux. Originally identified in Methicillin-Resistant *Staphylococcus aureus* (MRSA) of clinical origin, *poxtA* has rapidly disseminated across various Gram-positive bacteria, most notably *Enterococcus faecium* and *Enterococcus faecalis*, and is commonly found on highly transferable plasmids and transposons, often linked with other resistance genes [49]. *erm(40)* encodes a 23S rRNA methyltransferase that methylates A2058 in the ribosomal RNA, conferring resistance to MLS antibiotics [50]. Originally identified in *Bacillus halodurans* from environmental samples, *erm(40)* has been reported in soil, wastewater, and dairy-farm microbiomes, often associated with spore-forming *Bacillus* species [51–53]. Detection of *poxtA* and *erm(40)* in raw-milk and dairy-farm metagenomes underscores the persistence of transferable and intrinsic resistance elements within environmental reservoirs, highlighting the role of dairy production systems as important interfaces for the circulation of multidrug-resistance genes between animal, environmental, and human microbial communities [54,55].

Additionally, we observed a high prevalence of resistance genes against heavy metals such as copper and zinc, consistent with findings from previous studies [56–58]. These trace elements are commonly used in the livestock industry as feed additives and antimicrobials, including in footbaths to prevent lameness [59]. However, heavy metal resistance genes often co-localize with ARGs, facilitating co-selection. For instance, zinc has been shown to select for MRSA due to the co-location of the methicillin resistance gene (*mecA*) and zinc resistance gene (*czrC*) within the SCCmec element. Similarly, the transferable copper-resistance gene (*tcrB*) has been linked to macrolide and vancomycin resistance in *Enterococci* via shared conjugative plasmids [60]. These findings underscore the need for judicious use of heavy metals in dairy farming, as excessive application may drive both metal and antibiotic resistance.

The detection of multiple virulence factors, including iroC, *hlyB*, *ybtP*, and *rtxB*, across both organic and conventional farm environments underscores the widespread distribution of pathogenicity-associated secretion and iron-acquisition systems within dairy farm microbiome. Unlike AMR genes, identifying these virulence factors in a relatively high concentration in milk samples indicates their environmental persistence. The *iroC* gene is part of the *iroA* gene cluster (iroBCDEN), which encodes components of the salmochelin siderophore system responsible for iron acquisition in Gram-negative bacteria, particularly *Escherichia coli*, *Salmonella* sp., and *Klebsiella* sp [61]. The *hlyB* gene encodes the ATP-binding cassette (ABC) transporter component of the type I secretion system responsible for α-haemolysin (HlyA) export in *Escherichia coli*, which facilitates host-cell lysis, inflammatory responses, and contributes to extraintestinal pathogenic *E. coli* (ExPEC) virulence [62,63]. The *rtxB* gene encodes the secretion ATPase of the RTX (Repeats-in-Toxin) family and mediates the translocation of large cytotoxins across the bacterial envelope via the type I secretion apparatus, a key virulence mechanism in *Vibrio* sp., *Kingella* sp., and ExPEC pathotypes [64]. In contrast, *clbP*, encoding a periplasmic peptidase involved in colibactin activation within the *pks* pathogenicity island, prevalent in Enterobacteriaceae, was uniquely identified in the organic farm [65,66]. This correlates with the high prevalence of enterobacterial members in the organic farm samples. Collectively, these findings suggest that, despite not identifying the high prevalence of known host microbes of these virulence factors, the prevailing horizontal gene transfer of these virulence factors among the dairy microbiome and the environmental persistence.

This study has several important limitations that should be considered when interpreting the findings. First, the inclusion of only one organic and one conventional farm means that all between-farm differences are confounded with farm-specific factors such as geographic location, herd size, herd genetics, and individual management history. Consequently, the observed similarities in microbiome and resistome composition between farms cannot be generalized as evidence that farm management practices have limited influence on microbial communities across all organic and conventional operations. Second, pooling heterogeneous sample types under farm-level categories for statistical comparison introduces ecological confounding, as microbial communities differ substantially across matrices. While per-sample-type comparisons partially address this limitation, mixed-effects models that explicitly account for the hierarchical and nested structure of the data would provide more robust inference. Third, the present study did not include analysis of metagenome-assembled genomes (MAGs) for the resistome, which would have enabled association of ARGs with specific bacterial hosts and investigation of genetic context. A MAG-based resistome analysis is currently underway. Fourth, the study did not include analysis with AMRFinderPlus, which may offer improved specificity for certain clinically relevant resistance genes compared to CARD. Finally, the cross-sectional design captures a single time point and cannot address temporal dynamics or seasonal variation in microbiome and resistome composition. Future studies incorporating farm-level replication, longitudinal sampling, and MAG-based resistome analysis will be necessary to validate and extend these preliminary findings.

In summary, within the two farms examined in this study, microbiome and resistome compositions were largely comparable between the organic and conventional operations. Instead, the strikingly uniform microbial and antimicrobial resistance gene profiles observed within farms highlight the substantial role of microbial interchange among interconnected ecological niches. Future research should integrate analyses of cow-associated microbiomes, including skin and gastrointestinal communities, to comprehensively elucidate the ecological and biological factors driving microbial dynamics and resistance gene dissemination in dairy production systems. Such holistic approaches will advance our understanding and inform targeted interventions for maintaining animal health, product quality, and public health safety

## Methods

### Study design

For this study, dairy farms from one organic and one conventional dairy system in Texas were enrolled. The farms were located 153 miles apart. Both operated as closed herds in confinement, feeding Total Mixed ration with supplemental grazing in organic system during summer season to meet the USDA Organic requirement. Sampling on both farms was conducted in May 2023 by trained personnel, including veterinarians and students of veterinary and animal sciences. The dairy herds on both farms consisted of Holstein cattle, and their feed was primarily based on corn silage with other ingredients added to formulate a ration to meet the biological requirement of cows at different milk production levels. Both farms maintained Holstein herds, with bulk tank somatic cell counts averaging 325 and 350 thousand in conventional and organic farms, respectively. The organic farm housed approximately 2,500 cows, whereas the conventional farm maintained a herd of around 500. Both farms featured free-stall barns with concrete floors and sand bedding that was topped up with fresh sands two times per week. The organic farm operated 10 freestall pens maintained at 100% stocking density, whereas the conventional farm operated 4 freestall pens at 110% stocking density. In accordance with organic regulations, cows on the organic farm obtained more than 30% of their feed from pasture during each summer grazing season, which lasted over 120 days. The conventional farm used a water flush system to clean the alleyways, while the organic farm employed a lane cleaning and transport system (honeyvac) for manure removal. At both farms, sand bedding was not recycled; instead, fresh sand was routinely added to replenish the stalls. The stalls were scrapped on alternate days when bringing the cows to the milking parlor at both farms. The organic farm used a double-17 rapid-exit parallel milking parlor, while the conventional farm used a double-24 system. Both farms utilized iodine-based predip solution sourced from the same commercial distributor and used teat-dip cups for post-dip application Additionally, both farms used a backflushing system for cluster disinfection. Parlor floors were cleaned with water hoses whenever feces were observed, and the walking areas were thoroughly flushed with water at the end of each milking session. None of the farms used sanitizing agents to clean parlor floors and stanchions. Both of the farm followed normal milking routine as recommended by National Mastitis Council, which focused on wearing gloves, pre-dipping, fore-stripping, thorough drying with individual cloth towels, attaching and adjusting units, and post-dipping. The towels were washed with a detergent at the farm and dry towels were used. The protocol was to use one towel per cow and change gloves frequently. Cows with clinical mastitis were separated and moved to a hospital pen after identification of clinical signs in the udder and visibly altered milk. Both farms had history of mastitis cases associated with E.coli, Staphylococcus aureus while Organic farm also had incidence of Mycoplasma associated mastitis. Sick animals were milked as a last group in both farms followed by the detailed cleaning of the milking system. The conventional dairy farm used blanket dry cow therapy with the antibiotic Cephalosporins to combat the mastitis during dry period. Although the organic herd typically met pasture intake requirements during the summer, no grazing occurred during the sampling period.

Sampling was carried out at identical locations within both farms, comprising a total of eight sample types broadly categorized into two groups. The first group included samples from the milking parlour area, such as cow teat swabs, milking mats, liners, and milk samples. The second group consisted of samples from non-milking areas, including the feed area, bedding, and water troughs. Specifically, 60 ml per sample grab of used bedding materials (SAN) were collected from the back one third of the 10% of the stalls (evenly spaced throughout the free stall barns). The bedding materials were pooled in sterile plastic bags. Pooled bedding materials were mixed thoroughly by rolling and shaking in sample bags [67]. Swab samples were taken from the inner surfaces of water troughs (WAT), the mats at the milking parlour (MAT), and the concrete surfaces in the feed area (FEA). To assess changes in the teat microbiome and resistome due to iodine dipping as predip solution, swabs were collected from cow teats before (TBD) and after (TAD) iodine application. Milk samples (MIL) were collected directly from the same cows into sterile 50 mL conical tubes. In total, 180 samples were collected from organic (n = 99) and conventional farms (n = 81) (Table 1). Nine samples were excluded from metagenomics sequencing due to low concentration of DNA, including five from organic farms [SAN (3), and MIL (2)] and four from conventional farms [SAN (3), and MIL (1)]. Therefore, a total of 171 samples (organic; n = 94 and conventional; n = 77) were sequenced for this study. All samples were collected aseptically and transported to the laboratory at –20 °C for further analysis.

**Table 1:** Number of samples collected and sequenced from each sampling location within the organic and conventional study farms. A total of 180 samples were collected across both farms, of which 9 were excluded prior to metagenomic sequencing due to insufficient DNA concentration, yielding 171 samples for paired-end sequencing on the DNBSEQ-G400 platform. No sample pooling was performed; each sample was individually extracted and sequenced.

	Organic farm			Conventional farm
Sample type	Collected (n)	Sequenced (n)	Collected (n)	Sequenced (n)
Teats before dipping (TBD)	12	12	10	10
Teats after dipping (TAD)	12	12	10	10
Liner (LIN) ^a^	14	14	12	12
Milking parlour mat (MAT)	6	6	6	6
Feed area (FEA)	9	9	9	9
Water trough (WAT)	18	18	8	8
Sand bedding (SAN) ^b^	14	11	14	11
Milk (MIL) ^c^	14	12	12	11
**Total**	**99**	**94**	**81**	**77**
Excluded (insufficient DNA)	5	–	4	–
**Grand total collected**	**99**			**81**

^a^Includes 2 liner-before-use baseline samples per farm (4 total).

^b^Includes 2 unused sand baseline samples per farm. Three organic and three conventional sand samples were excluded due to insufficient DNA concentration.

^c^Includes individual cow milk and bulk tank milk samples. Two organic and one conventional milk sample were excluded due to insufficient DNA concentration.

The organic farm contributed more samples owing to its larger herd size (~2,500 vs. ~ 500 cows) and greater number of freestall pens (10 vs. 4), which required broader spatial sampling.

TBD, teats before dipping; TAD, teats after dipping; LIN, liner; MAT, milking parlour mat; FEA, feed area; WAT, water trough; SAN, sand bedding; MIL, milk.

All animal handling and sampling protocols for this study were approved by the Texas A&M AgriLife Animal Care and Use Committee [AACUC #2022-026A].

### DNA extraction and metagenomic sequencing

Genomic DNA extraction from all swab samples was performed using the DNeasy PowerSoil Pro Kit (Qiagen, Hilden, Germany), while DNA extraction from milk samples was conducted using the DNeasy Blood & Tissue Kit (Qiagen, Hilden, Germany), following the manufacturer’s instructions. DNA libraries were prepared according to the MGIEasy Universal DNA Library Prep Set User Manual v1 Protocol (MGI Tech Co., Shenzhen, China). This process involved fragmenting genomic DNA using the Covaris M220 Focused-Ultrasonicator (Covaris, Brighton, UK), followed by end repair and A-tailing of the sheared DNA. The resulting sheared DNA was used to create DNA Nanoballs (DNBs), which were sequenced using the DNBSEQ-G400 sequencer (MGI Tech Co., Shenzhen, China) in paired-end mode (PE150), following the manufacturer’s guidelines. The target sequencing depth was approximately 24 million read pairs (~7.2 Gb) per sample.

### Bioinformatics analysis

#### Quality control.

Sequencing reads were checked for barcodes and adapter sequences using Trimmomatic v0.39 with the following parameters: low-quality bases (Q < 20) were trimmed using a 4-base sliding window, bases with quality scores below 10 were trimmed from both the leading and trailing ends and reads shorter than 60 bp after trimming were discarded. [68]. Subsequently, the raw reads from each sample were processed to remove low-quality sequences using default parameters, followed by quality control assessment with FastQC v0.11.9 [69]. Depletion of host reads were carried out by aligning against *Homo sapiens* GRCh38 reference assembly through Bowtie2 v2.5.4 in strict mode. The unaligned reads were again aligned with *Bos taurus* reference assembly (NCBI RefSeq Accession No.: NW_020191524.1) using Bowtie2 v2.5.4 in strict mode. The quality-checked, host contamination removed reads were processed using two approaches: (i) reference-based and (ii) assembly-based.

#### Reference-based module.

Species composition and diversity of the samples were assessed using Kraken2 v2.1.4 [70], which employs an exact k-mer matching classification system for high accuracy and rapid taxonomic classification. The classification was carried out against the Kraken 2 standard database updated June 2023. The standard database is based on the taxonomic information from RefSeq from archaea, bacteria, fungi, viruses, protozoa, known vectors and human sequences.Taxonomic classification and abundance were calculated at seven levels: Kingdom, Phylum, Class, Order, Family, Genus, and Species. For the analysis of ESKAPE pathogen composition, a subset of the data was rescaled to reflect the combined abundance of *Enterococcus faecium*, *Staphylococcus aureus*, *Klebsiella pneumoniae*, *Acinetobacter baumannii*, *Pseudomonas aeruginosa*, and *Enterobacter* spp. [71]. The relative abundance of ARGs within the metagenomic datasets was identified by aligning the sequences against the Comprehensive Antibiotic Resistance Database (CARD) v3.2.9 [72] using BLASTN (evalue: 1e-5; perc_identity: 90; -qcov_hsp_perc: 90). For ARG normalization and abundance estimation, the number of 16S rRNA gene copies present in each sample was determined using METAXA2 v2.2 [73].

#### Assembly-based module.

Clean reads were individually assembled using MEGAHIT v1.2.9 with contigs length ≥ 1000 bp [74]. Open reading frames (ORFs) were predicted using Prodigal v2.6.3 in meta mode [75]. Redundant ORFs were removed using CD-HIT v4.8.1 with 90% identity, 90% coverage, and a length of 250 bp [76]. Unique ORFs were aligned with CARD databases using DIAMOND v2.1.10 with > 70% similarity, e-value = 10 ^−10^, and > 50% query coverage [77]. Following the analysis the hits were analyzed based on the CARD database ontology. The hits were classified at gene family level followed by drug class and resistance mechanism. The normalized abundance of ARGs at class level was expressed as ‘copy of ARG per copy of 16S-rRNA gene’ determined using the following formula,


$$\begin{array}{c} Abundance=\sum_{\text{1}}^{n}\frac{N_{ARG-likesequence}XL_{reads}/L_{ARGreferencesequence}}{N_{\text{1}\text{6}Ssequence}XL_{reads}/L_{\text{1}\text{6}Ssequence}} \end{array}$$

where N_ARG_-like sequence is the number of the ARG-like sequence annotated as one specific ARG reference sequence; L_ARG_ reference sequence is the sequence length of the corresponding specific ARG reference sequence; N_16S_ sequence is the number of the 16S sequence identified from the metagenomic data; L_16S_ sequence is the average length of the 16S sequence in the Greengenes database, which was used as the reference database for the 16S sequence that is, 1432 bp n is the number of the mapped ARG reference sequence belonging to the ARG type or subtype; L_reads_ is the sequence length of the DNBSEQ-G400 reads (300 nt) that was used in the present study [78].

The risk ranking v3.0 of ARGs prevalent in the dairy samples was determined based on a previously established risk-ranked ARG list provided by the SARG database. This ranking was originally developed by analyzing a comprehensive collection of global metagenomes (n = 1,427) and the RefSeq genome database, covering 114,703 reference ARGs. The risk classification of ARGs was based on three key criteria: association with human environments, mobility, and presence in pathogenic hosts. ARGs were ranked based on their enrichment in human-associated environments, mobility, and presence in pathogenic hosts. Those with <100-fold enrichment in human environments were classified as Rank IV (lowest risk). Among the enriched ARGs, non-mobile ones were assigned Rank III; mobile ARGs not found in pathogens were Rank II; and mobile ARGs enriched in human environments and present in pathogens were assigned the highest risk, Rank I [79].

Further, the prevalence of intergrase, heavy-metals resistance genes, and virulence factors by performing the sequence alignment against INTEGRALL v1.2 [80], BacMet v2.0 [81], and VFDB [82] respectively, through DIAMOND v2.1.10 using the same parameters. The presence of plasmids within the contigs was determined through MOB-Recon v3.1.9 and the taxonomy of the plasmids was identified through after the carrying out sequence similarity search against the inbuilt database originally constructed from the NCBI Refseq plamids [83]. The ARGs identified within the plasmid sequence were identified with BLASTN (e-value cutoff: 0.001) through the same parameters as described above by aligning against CARD database.

### Statistical analysis

Parametric data were statistically compared using Student’s t-test and one-way analysis of variance (one-way ANOVA), while data with nonparametric distribution were compared with nonparametric Wilcoxon rank-sum test or Kruskal-Wallis test. All p-values were adjusted for multiple comparisons using Benjamini–Hochberg false discovery rate (FDR) correction, and an adjusted p-value of < 0.05 was considered significant. For beta-diversity analysis, we conducted Principal Coordinate Analysis (PCoA), and Permutational Multivariate Analysis of Variance (PERMANOVA) was applied to compare clustering ordination with 999 permutations and homogeneity of multivariate dispersions (PERMDISP) was also applied to test for evaluating the differences in within-group variability. Linear discriminant analysis effect size (LEfSe) was used to identify differentially abundant microbiome taxa and ARGs, implemented in the MicrobiomeAnalyst platform. A log LDA score threshold of >4 was applied to focus on taxa and genes with large effect sizes representing biologically meaningful differences, a more stringent cutoff than the default threshold of >2, as recommended for metagenomic datasets with large numbers of features (Segata et al., 2011). Positive and negative interactions among the bacterial genera and ARGs were identified through SparCC (Sparse Correlations for Compositional data) [84] SparCC addresses the limitations of Pearson and Spearman-rank correlation methods by estimating correlations that account for the compositional structure of the data. It applies log-ratio transformations to correct for proportionality effects, thereby minimizing the biases inherent in conventional correlation measures. The interactions were computed as the mean of 50 iterations, and pseudo-p values were estimated through 100 bootstrap resamplings. Correlations with r ≥ 0.6 and p ≤ 0.05 were considered significant for downstream analyses. Further, the shared microbiome between the various samples were identified with UpSet venn plots [85] The significance of association among the microbiomes and the ARGs, integrons, and metal resistance genes were assessed with Mantel test with 999 permutations, performed using the vegan package (2.6–10) in R. Additionally, a Procrustes analysis with 999 permutations conducted with vegan package (2.6–10) to further evaluate the similarity between the two distance matrices. The Procrustes M² value, representing the sum of squared distances between matched sample pairs, was used as a measure of fit. To evaluate the potential confounding effect of sample matrix on farm-level comparisons, PERMANOVA was additionally performed after excluding milk samples (n = 148) and with sampling location as a covariate. Per-sample-type alpha diversity comparisons between farm types were conducted using Mann-Whitney U tests within each of the eight sample types, with Benjamini-Hochberg FDR correction applied across comparisons.

## Supporting information

S1 Fig Alpha diversity metrics representing the overall microbiome distribution in organic and conventional farms: (A) ACE, (B) Chao1 index. (TIFF)

S2 Fig Alpha diversity measures – (A) ACE, (B) Chao1 index of the microbiome distribution observed among the various samples obtained from the organic and conventional farms. FEA -Feed area, LIN – Liners, MAT- Mat, MIL – Milk, SAN – Sand, TAD – Teats after iodine dipping, TBD – Teats before iodine dipping, WAT – Water troughs.
(TIFF)

S3 Fig Stacked bar charts displaying the relative abundance of bacterial phyla of the microbiome obtained from sampling sites from organic and conventional farms. FEA -Feed area, LIN – Liners, MAT- Mat, MIL – Milk, SAN – Sand, TAD – Teats after iodine dipping, TBD – Teats before iodine dipping, WAT – Water troughs.
(TIFF)

S4 Fig Linear discriminant analysis effect size (LEfSe) identifying bacterial genera significantly enriched in samples from conventional and organic farms. (JPEG)

S5 Fig UpSet Venn plots showing the common bacterial genera shared among the various sampling sites across the (A) conventional and (B) organic farms. FEA -Feed area, LIN – Liners, MAT- Mat, MIL – Milk, SAN – Sand, TAD – Teats after iodine dipping, TBD – Teats before iodine dipping, WAT – Water troughs.
(JPEG)

S6 Fig Bar plot illustrating the relative abundance of antimicrobial resistance genes (ARGs) categorized by resistance mechanism in samples from organic and conventional dairy farms. FEA -Feed area, LIN – Liners, MAT- Mat, MIL – Milk, SAN – Sand, TAD – Teats after iodine dipping, TBD – Teats before iodine dipping, WAT – Water troughs.
(PNG)

S7 Fig Bar plot illustrating the relative abundance of antimicrobial resistance genes (ARGs) grouped by drug class in samples from organic and conventional dairy farms. (PNG)

S8 Fig Linear discriminant analysis effect size (LEfSe) identifying ARGs significantly enriched in samples from conventional and organic farms. (PNG)

S9 Fig UpSet Venn plots showing the common ARGs shared among the various sampling sites across the (A) conventional and (B) organic farms. FEA -Feed area, LIN – Liners, MAT- Mat, MIL – Milk, SAN – Sand, TAD – Teats after iodine dipping, TBD – Teats before iodine dipping, WAT – Water troughs.
(JPEG)

S10 Fig Volcano plot of differential abundance analysis (ANCOM-BC) between organic and conventional dairy farm samples. Each point represents one genus (n = 2,710 tested). The x-axis shows log2 fold-change (organic/conventional) and the y-axis shows -log10(q-value). Blue points indicate genera significantly enriched in organic farm samples (n = 416, q < 0.05); red points indicate genera significantly enriched in conventional farm samples (n = 412, q < 0.05); grey points indicate non-significant genera (n = 1,882). The dashed horizontal line indicates the significance threshold (q = 0.05). Key genera are labeled.
(PNG)

S11 Fig Top 30 differentially abundant genera between organic and conventional dairy farms identified by ANCOM-BC (q < 0.05). Blue bars indicate genera with higher abundance in organic farm samples; red bars indicate genera with higher abundance in conventional farm samples. Error bars represent standard errors of the log2 fold-change estimates. Genera are ranked by absolute log2 fold-change.
(PNG)

S1 Table Assembly statistics of the metagenomic datasets generated in this study. (XLSX)

S2 Table List of metal resistance genes obtained after sequence alignment against BacMet database. (XLSX)

S3 Table List of integrons obtained after sequence alignment against integrall database. (XLSX)

S4 Table List of virulence factors obtained after sequence alignment against VFDB database. (XLSX)

S5 Table Aggregate MOB-typer report for all contigs obtained after plasmid prediction in metagenomic datasets. (XLSX)
