# Supplementary figures and images for: Insights into spatial dynamics of the microbiome and resistome across the conventional and organic dairy farms
Source: PLoS One. 2026 Jun 25;21(6):e0352336. doi: 10.1371/journal.pone.0352336 (PMC13298916; doi:10.1371/journal.pone.0352336)

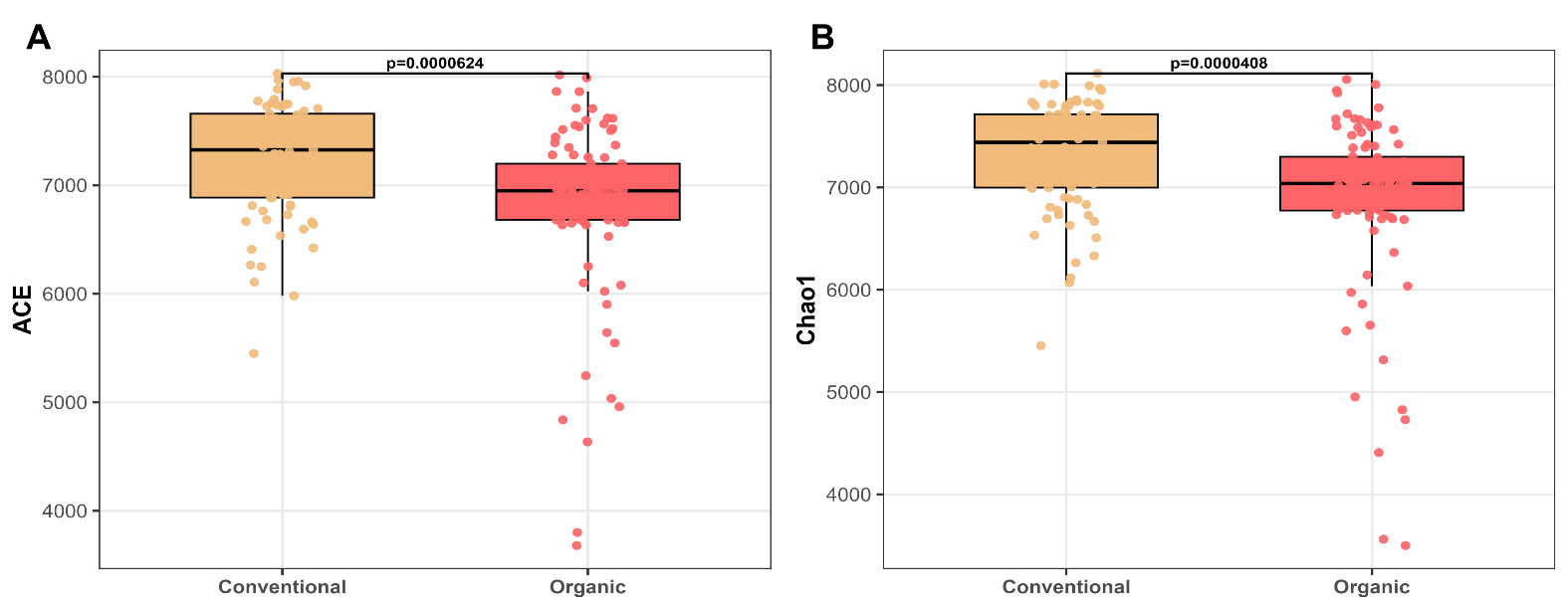

Supplement: S1 Fig — (TIFF) [file pone.0352336.s001.tiff]

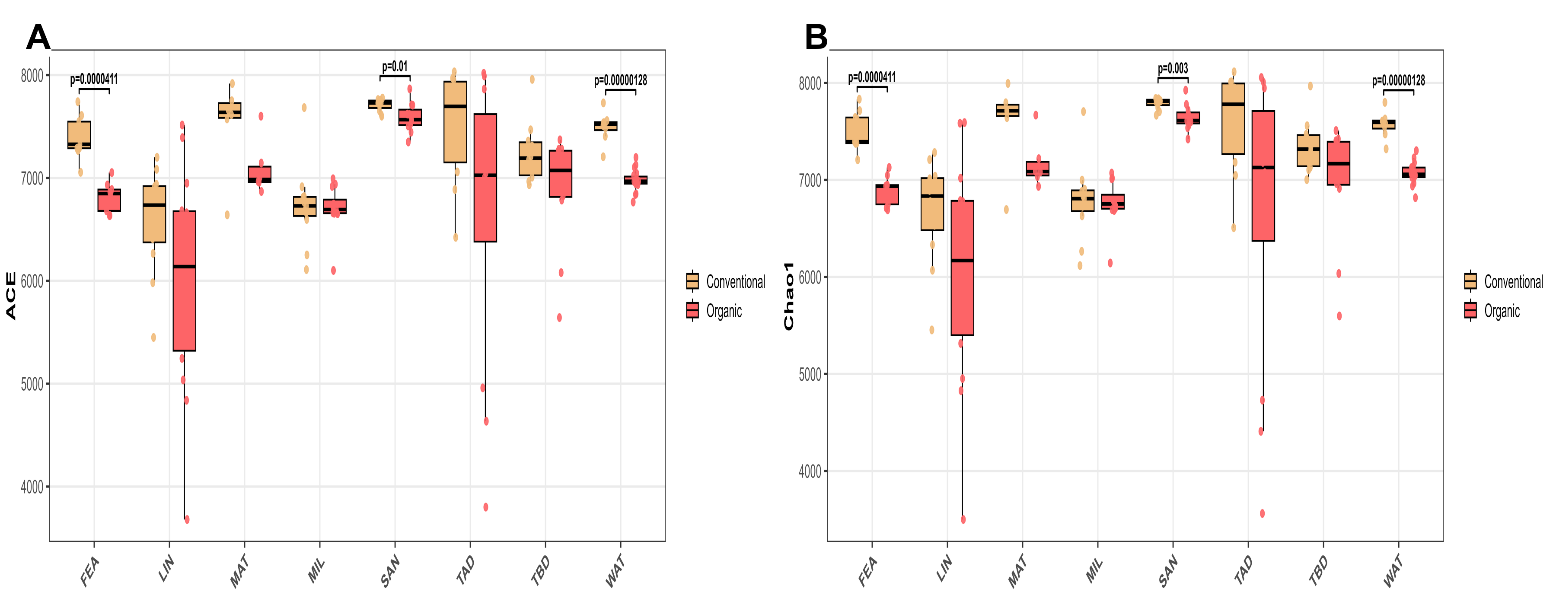

Supplement: S2 Fig — FEA -Feed area, LIN – Liners, MAT- Mat, MIL – Milk, SAN – Sand, TAD – Teats after iodine dipping, TBD – Teats before iodine dipping, WAT – Water troughs. (TIFF) [file pone.0352336.s002.tiff]

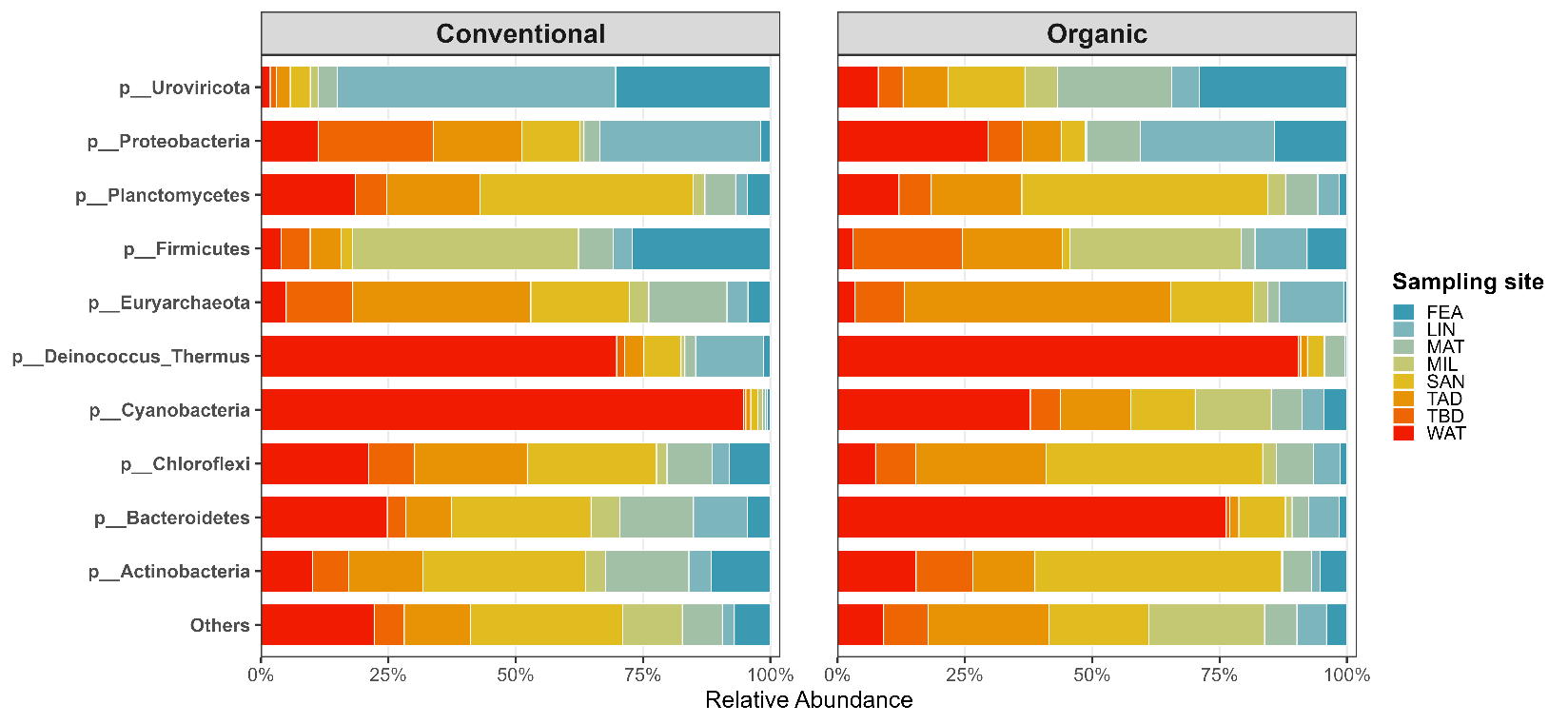

Supplement: S3 Fig — FEA -Feed area, LIN – Liners, MAT- Mat, MIL – Milk, SAN – Sand, TAD – Teats after iodine dipping, TBD – Teats before iodine dipping, WAT – Water troughs. (TIFF) [file pone.0352336.s003.tiff]

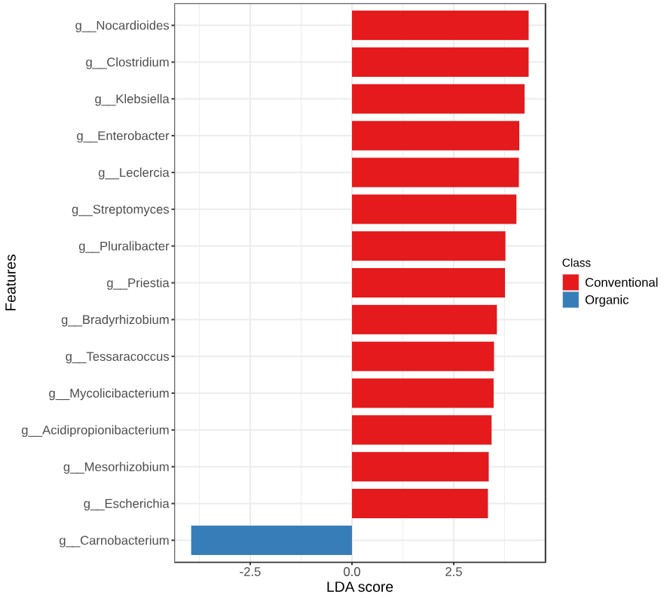

Supplement: S4 Fig — (JPEG) [file pone.0352336.s004.jpeg]

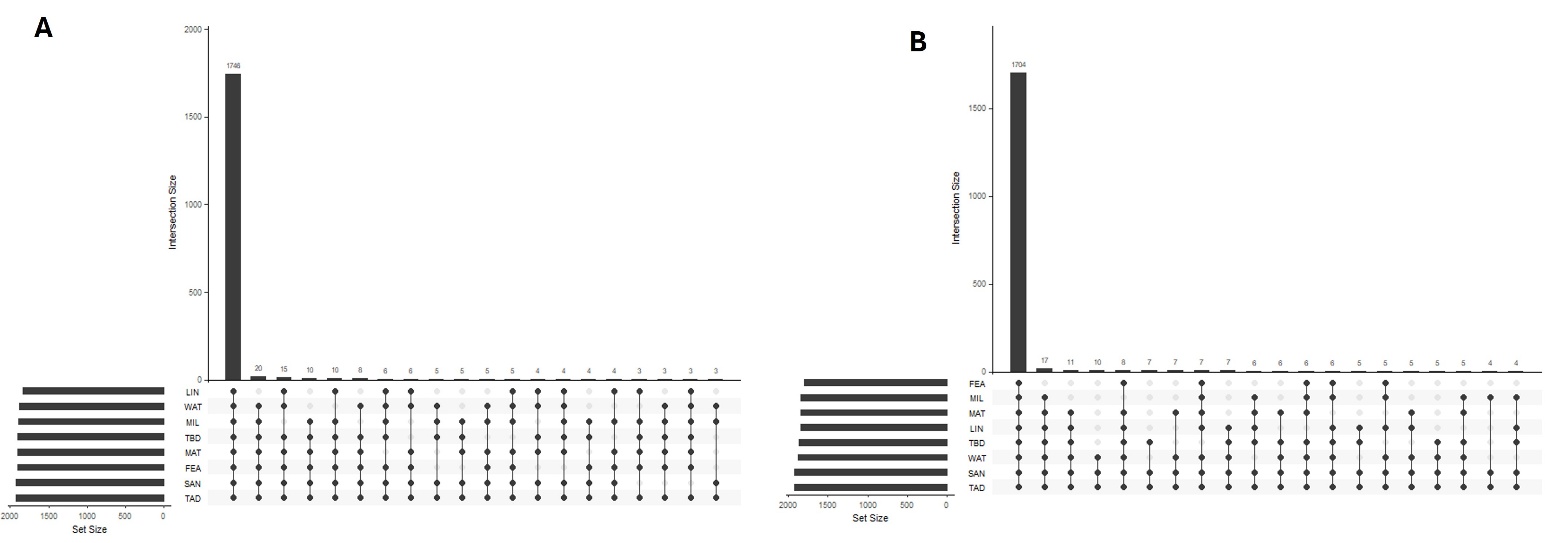

Supplement: S5 Fig — FEA -Feed area, LIN – Liners, MAT- Mat, MIL – Milk, SAN – Sand, TAD – Teats after iodine dipping, TBD – Teats before iodine dipping, WAT – Water troughs. (JPEG) [file pone.0352336.s005.jpeg]

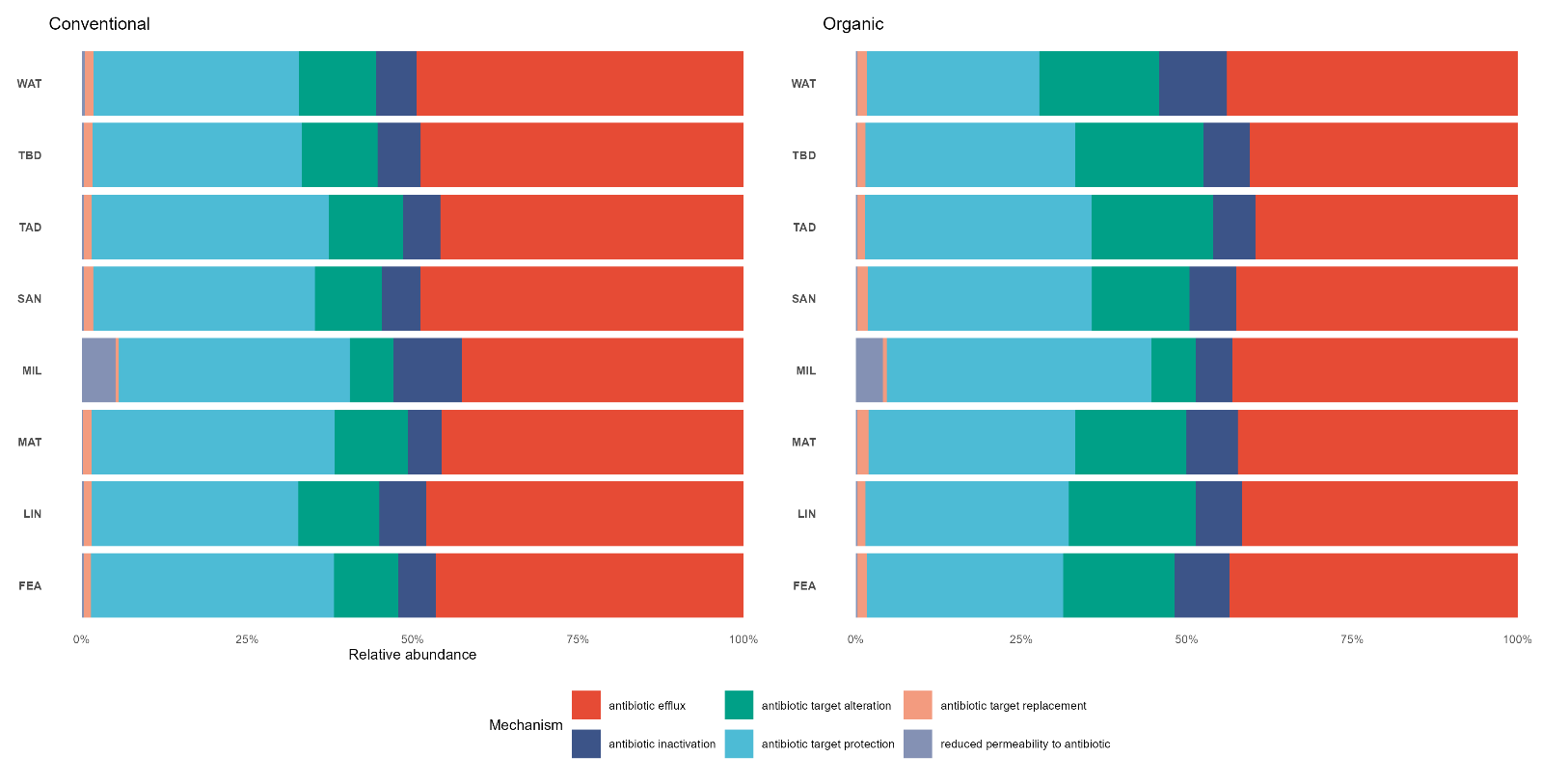

Supplement: S6 Fig — FEA -Feed area, LIN – Liners, MAT- Mat, MIL – Milk, SAN – Sand, TAD – Teats after iodine dipping, TBD – Teats before iodine dipping, WAT – Water troughs. (PNG) [file pone.0352336.s006.png]

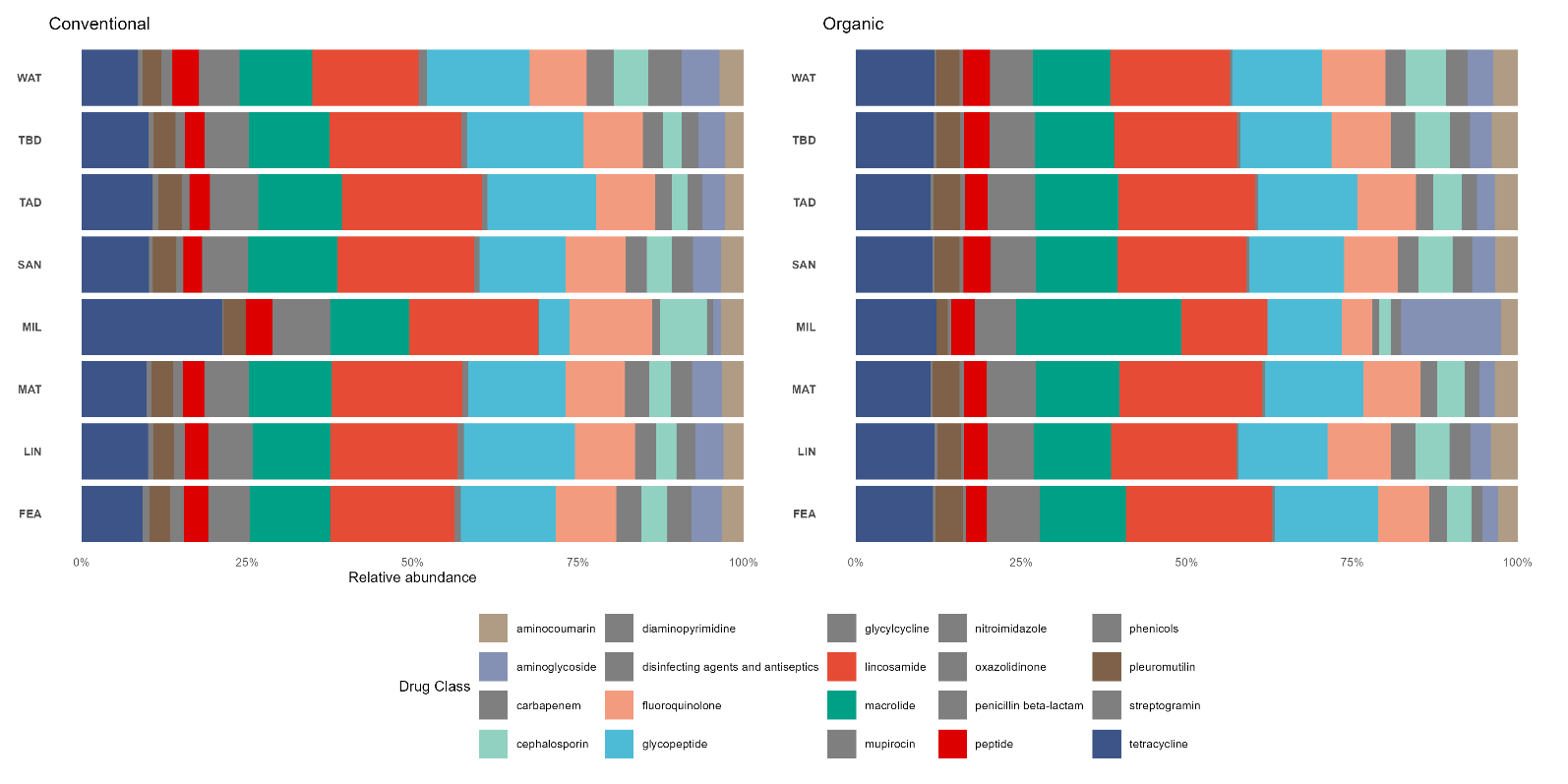

Supplement: S7 Fig — (PNG) [file pone.0352336.s007.png]

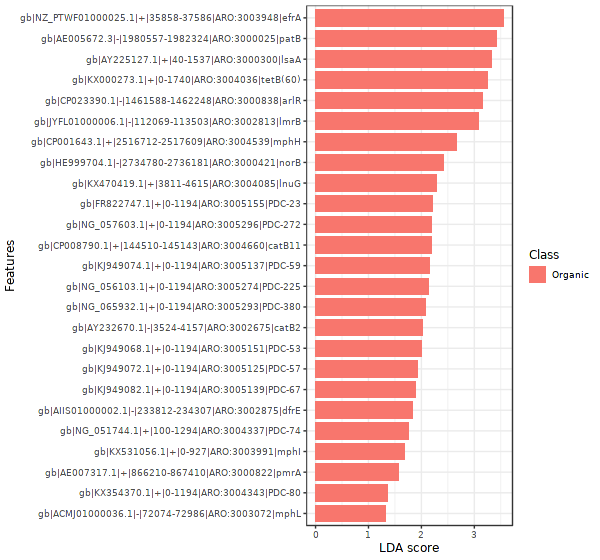

Supplement: S8 Fig — (PNG) [file pone.0352336.s008.png]

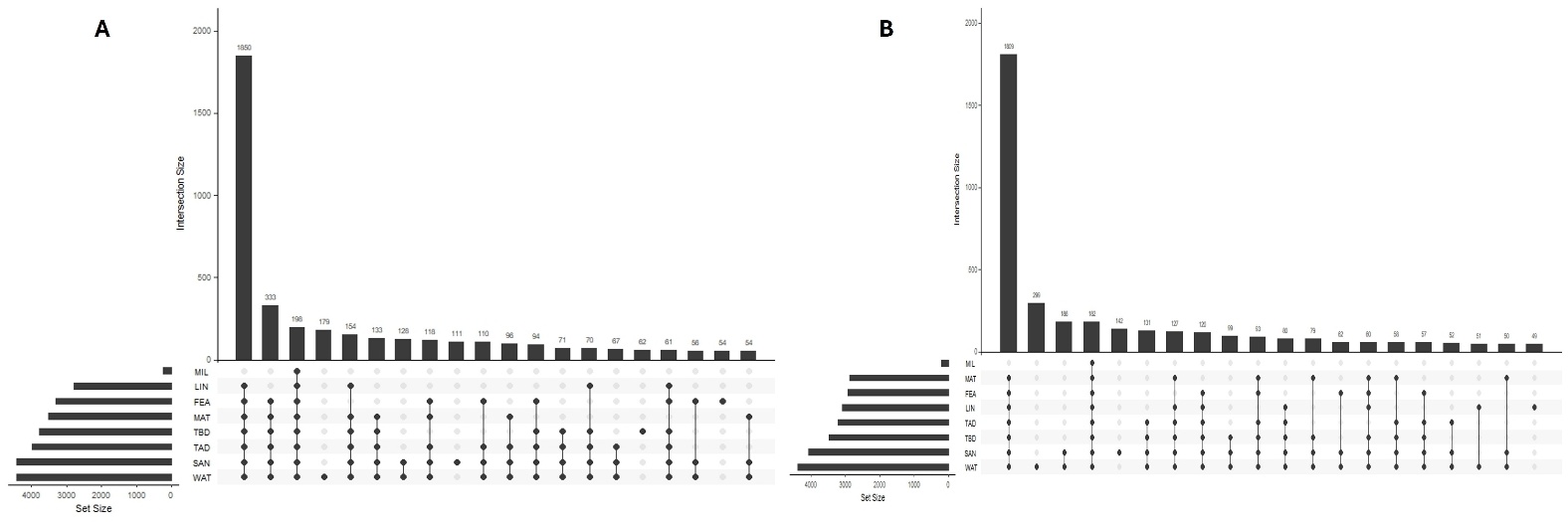

Supplement: S9 Fig — FEA -Feed area, LIN – Liners, MAT- Mat, MIL – Milk, SAN – Sand, TAD – Teats after iodine dipping, TBD – Teats before iodine dipping, WAT – Water troughs. (JPEG) [file pone.0352336.s009.jpeg]

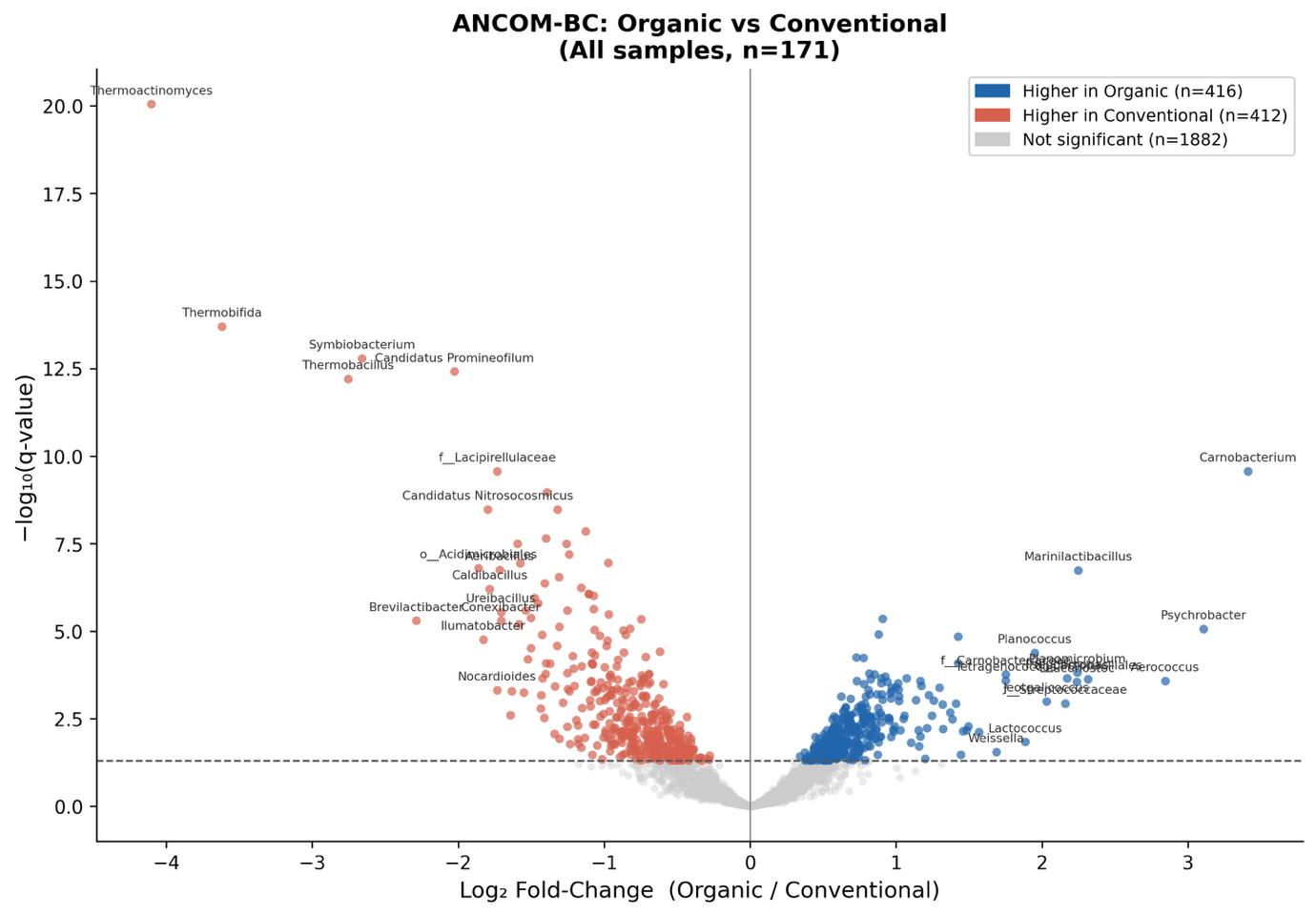

Supplement: S10 Fig — Each point represents one genus (n = 2,710 tested). The x-axis shows log2 fold-change (organic/conventional) and the y-axis shows -log10(q-value). Blue points indicate genera significantly enriched in organic farm samples (n = 416, q < 0.05); red points indicate genera significantly enriched in conventional farm samples (n = 412, q < 0.05); grey points indicate non-significant genera (n = 1,882). The dashed horizontal line indicates the significance threshold (q = 0.05). Key genera are labeled. (PNG) [file pone.0352336.s010.png]

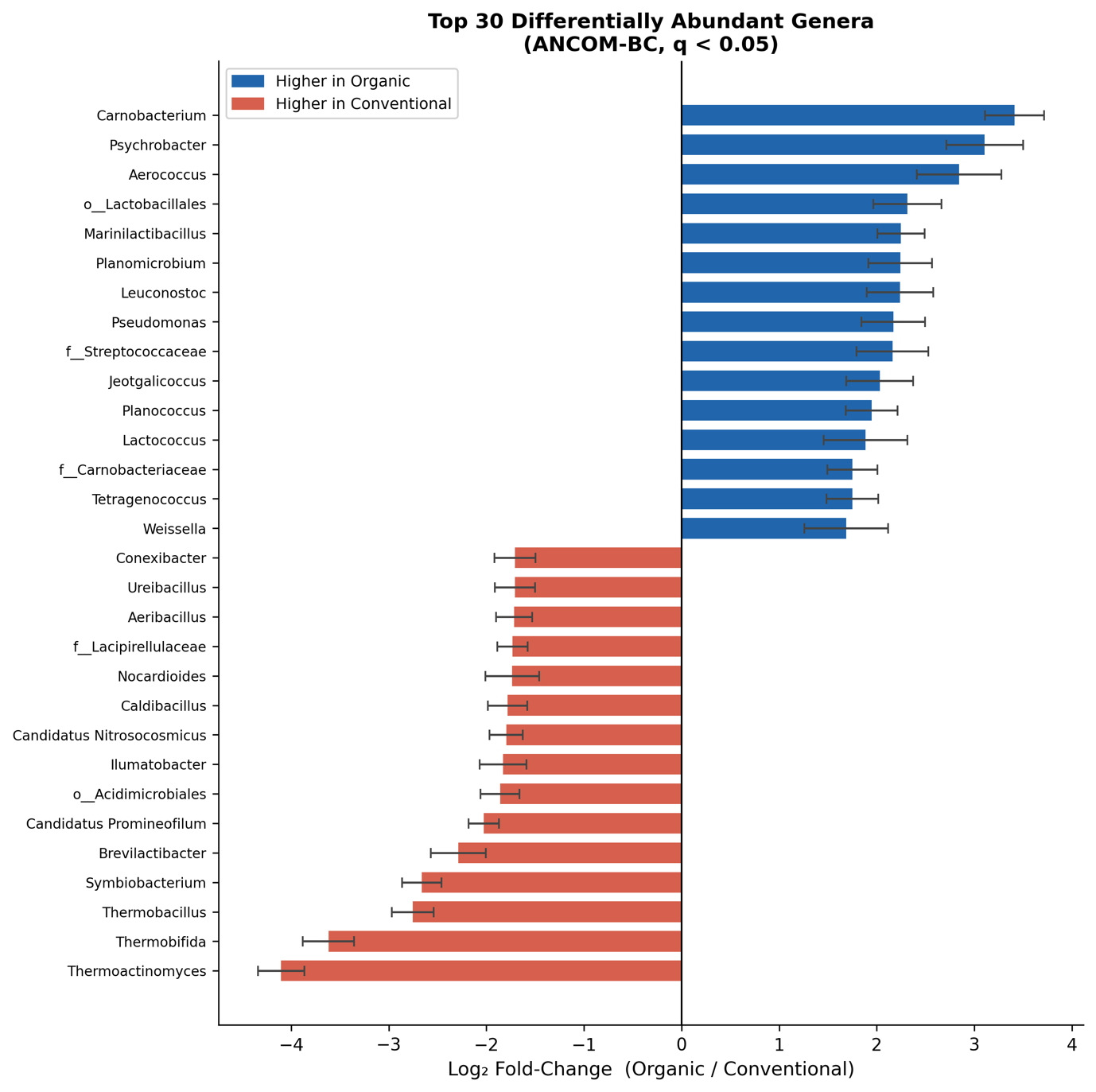

Supplement: S11 Fig — Blue bars indicate genera with higher abundance in organic farm samples; red bars indicate genera with higher abundance in conventional farm samples. Error bars represent standard errors of the log2 fold-change estimates. Genera are ranked by absolute log2 fold-change. (PNG) [file pone.0352336.s011.png]
